# Age-related differences in gait adaptations during overground walking with and without visual perturbations using a virtual reality headset

**DOI:** 10.1038/s41598-020-72408-6

**Published:** 2020-09-21

**Authors:** Muyinat Y. Osaba, Dario Martelli, Antonio Prado, Sunil K. Agrawal, Anil K. Lalwani

**Affiliations:** 1 Columbia University Vagelos College of Physicians and Surgeons, New York, NY USA; 2 Department of Mechanical Engineering, University of Alabama, Tuscaloosa, AL USA; 3 Department of Mechanical Engineering, Columbia University, New York, NY USA; 4 Department of Rehabilitation and Regenerative Medicine, Columbia University Vagelos College of Physicians and Surgeons, New York, NY USA; 5 Department of Otolaryngology-Head and Neck Surgery, Columbia University Vagelos College of Physicians and Surgeons, 180 Fort Washington Avenue, Harkness Pavilion, 8th Floor, New York, NY 10032 USA; 6 New York Presbyterian Hospital, New York, NY USA

**Keywords:** Biomedical engineering, Geriatrics, Rehabilitation

## Abstract

Older adults have difficulty adapting to new visual information, posing a challenge to maintain balance during walking. Virtual reality can be used to study gait adaptability in response to discordant sensorimotor stimulations. This study aimed to investigate age-related modifications and propensity for visuomotor adaptations due to continuous visual perturbations during overground walking in a virtual reality headset. Twenty old and twelve young subjects walked on an instrumented walkway in real and virtual environments while reacting to antero-posterior and medio-lateral oscillations of the visual field. Mean and variability of spatiotemporal gait parameters were calculated during the first and fifth minutes of walking. A 3-way mixed-design ANOVA was performed to determine the main and interaction effects of group, condition and time. Both groups modified gait similarly, but older adults walked with shorter and slower strides and did not reduce stride velocity or increase stride width variability during medio-lateral perturbations. This may be related to a more conservative and anticipatory strategy as well as a reduced perception of the optic flow. Over time, participants adapted similarly to the perturbations but only younger participants reduced their stride velocity variability. Results provide novel evidence of age- and context-dependent visuomotor adaptations in response to visual perturbations during overground walking and may help to establish new methods for early identification and remediation of gait deficits.

## Introduction

Impaired balance and gait are associated with an increased risk of falls and significant morbidity and mortality in those aged 65 and older^1^.

Early detection of higher risk for falls is critical to begin timely intervention prior to fall episodes^2^. Gait assessments are used extensively to identify those at higher risk for falling^3^. Traditional gait assessments are principally based on volitional movements. However, most falls occur when an individual fails to recover from an unexpected loss of balance^4^. Recovering from gait perturbation elicits very specific neural control strategies and people at higher risk for falling usually show a reduced ability to recover^5,6^. Therefore, gait assessments that challenge balance have been proposed as a more effective method for early identification and remediation of gait deficits^7,8^. The majority of research performed using simulated balance-demanding tasks have included mechanical perturbations using either moveable platforms, obstacles, slippery surfaces, treadmill accelerations, and waist-pull perturbations^5,7,8^.

Another approach is to use sensory perturbations in the form of visual perturbations through immersive Virtual Reality (VR) systems. Stability and balance during walking is achieved through the integration of visual, vestibular, proprioceptive and other sensory inputs^9,10^. Increases in falls in the elderly may be related to the decline in visual capacity, specifically related to their difficulty in integrating multi-sensory information during complex motor tasks and their poorer perceptions of moving visual stimuli^9,11^. Balance in older adults tends to be more affected by altered visual input than balance in younger adults^12,13^, and older adults have increased reliance on visual feedback for control of balance during gait^14,15^. Indeed, they weigh visual stimuli higher than younger adults when maintaining posture during exposure to visual perturbations while older adults who are at a higher risk of falls weigh visual stimuli even higher than healthy older adults^16^. In addition to being more reliant on visual stimuli to maintain balance, older adults tend to adapt slower and incompletely to new or inaccurate visual information^11,13,15–17^. This may be due to difficulty when making appropriate on-line adjustments and re-weighting of the available sensory information to changing optic flows^11^.

Recent advancements in virtual reality (VR) technology have allowed access to novel paradigms for studying balance. Specifically, VR provides a safe and controlled environment for studying how humans react and adapt to the virtual environment (VE) and to conflicting sensorimotor stimulation^18^. VR systems that include sensory discordance in terms of unpredictable oscillations of the visual field can challenge an individual’s ability to maintain stability, thus targeting the neuromuscular skills required for fall prevention. Studying the effect of visual perturbations during gait allows for understanding the ways in which individuals respond to challenges to their balance and may be useful for diagnostic and training purposes.

Studies that analyzed gait in response to visual perturbations have mostly been conducted using treadmills rather than overground walking with virtual screens and domes rather than virtual reality headsets. Virtual reality headsets, however, have many potential advantages including: (1) providing immersive binocular depth cues and a first-person point of view for better responses to visual stimuli, (2) increasing the area through which a user can navigate which allows for overground-based rather than treadmill-based interventions, and (3) allowing for comparatively cheaper and portable exposure to the virtual environment in comparison with virtual reality domes or screens^18,19^.

Previous studies have showed that young adults walking on treadmills in the virtual environment display a shorter stride length, increased step width, and increased variability in stride velocity and step width in comparison to walking in the real environment^20^. Furthermore, during treadmill walking, young adults have increased gait instability when exposed to anteroposterior (AP) or mediolateral (ML) perturbations but are more affected by ML perturbation than the AP perturbation, with increased instability in the direction of the perturbation^21^. Exposure to visual perturbations during treadmill walking is also associated with shorter step length and increased step width compared to the unperturbed state, with increased effect of ML perturbations compared to AP perturbations. ML and AP visual perturbations are also associated with increased variability in step width and step length compared to the unperturbed state, while ML visual perturbations are associated with increased variability in step width and stride length compared to AP visual perturbations. ML perturbations are associated with increased stride time variability and decreased stride time compared to the unperturbed state^22^.

Similarly, in older adults, walking in the VE on a treadmill is associated with a temporary decrease in stride length and a sustained increase in step width^23^. Although increased step length variability is temporary, step width variability shows a sustained increase. Visual perturbations affect gait in older adults more than healthy young subjects^14,24^ thus highlighting their increased reliance on visual feedback to maintain balance. Older adults walk with shorter step length and greater step width, step length, and hip joint angle variability in comparison to young adults^25^. In contrast to younger adults who show increased hip joint variability in the coronal plane only when exposed to ML visual perturbations, older adults not only show increased hip joint variability in the coronal plane but also increased hip, knee, and ankle variability in the sagittal plane and of larger magnitude for any given visual perturbation^25^. Similarly, in comparison to young adults, older adults display a significantly larger ML sway and increased local instability when walking with ML visual perturbation^14^. Although exposure to ML visual perturbations is associated with increased variability of step width^24–26^, variability of step length^24,26^, and increased step width in both young and old adults^26^, ML perturbations are associated with shortened steps in older adults only^24–26^. Older adults also show more step width variability in response to ML perturbations when compared to young subjects, suggesting decreased control of lateral step placement^14^.

Although these studies provided insight into age-related changes in gait due to the VE and multidirectional visual perturbations, they have been done on treadmills rather than during overground walking. Motorized treadmills may produce erroneous or misleading results, particularly in situations where changes in neuromuscular control are likely to impact the variability or stability of locomotion, such as in older people^27^. Indeed, although older adults show evidence of familiarization with treadmill walking and similar walking patterns between overground walking and treadmill walking with increased time spent with treadmill walking in the VE, they continue to walk with a more cautious gait during treadmill walking compared to overground walking, even after 20 min of familiarization^28^. Moreover, these studies also do not focus on changes in gait parameters with exposure to visual perturbations over time, a possible indicator of gait adaptability. The ability to adapt to visual perturbations would demonstrate that older adults are still able to re-weight sensory stimulus and modify their ability to control balance while walking, which could be beneficial to implement preventive and rehabilitation programs targeting fall risk in older adults.

We have recently developed a VR platform that allows us to analyze the effect of visual perturbations and the prevalence of visuomotor adaptation during unperturbed overground walking or in response to continuous multidirectional visual oscillations in a VR headset^18^. A study conducted on 12 young participants showed that overground walking in the VE was associated with shorter stride length and higher stride width variability compared to the real environment (RE). When exposed to visual perturbations, young adults displayed decreased stride length, increased stride width, and increased stride variability, particularly with visual perturbations in the ML direction. However, they appeared to adapt to both the VE and the perturbations by increasing stride length and decreasing stride width, stride time variability, stride length variability, and stride time^18^. The aims of this pilot study were to investigate age-related modifications and propensity for visuomotor adaptations over time of spatio-temporal gait parameters due to prolonged walking in a VE, and during continuous antero-posterior (AP) and medio-lateral (ML) oscillations of the visual field. We hypothesize that: (1) the spatiotemporal gait characteristics of older adults will be more affected by the virtual environment and visual perturbations when compared to younger adults, (2) older adults will adapt to the virtual environment and visual perturbations but to a lesser degree than young adults, and (3) effects would be larger for ML perturbations than AP perturbations.

## Methods

Twelve young and twenty-six older subjects participated in the experiment. The older subjects consisted of adults who visited the Otolaryngology Clinic at Columbia University Irving Medical Center while the younger adults were healthy volunteers recruited at Columbia University’s undergraduate campus. Data for young participants was the same used in our previous study^18^. All methods/experiments were carried out in accordance with relevant guidelines and regulations. All participants signed a written informed consent form. Research received ethical approval from Columbia University’s Institutional Review Board prior to subject participation. All subjects had normal or corrected-to-normal vision. Given that we were interested in older adults without diagnosed vestibular or neurological disorders who ambulated independently, the following subjects were excluded: four subjects with a diagnosis of vertigo, one patient who walked with a cane, and one patient with a history of Parkinson’s disease. Thus, twenty older subjects and twelve young subjects were included in data analysis. Table 1 summarizes anthropometric characteristics for the twenty elderly and twelve young subjects included in the study.

**Table 1:** Subjects’ anthropometric characteristics.

	Old subjects (n = 20)	Young subject (n = 12)
Age [yrs]	79.6 ± 6.4	24.8 ± 3.9
Gender [% female]	55	33
Height [cm]	167.2 ± 11.4	173.7 ± 9.1
Body mass [kg]	70.6 ± 16.1	74.9 ± 14.9

Values represent mean ± one standard deviation.

The protocol below is the same of that described in Martelli et al.^18^ Subjects were instructed to walk for either 5 min (older adults) or 6 min (younger adults) on a 6.0 m × 0.6 m instrumented walkway (Zeno Walkway, Protokinetics) at their self-selected speed under four conditions: real environment (RE), virtual environment (VE), virtual environment with medio-lateral (ML) visual perturbations, and virtual environment with antero-posterior (AP) visual perturbations. A 1-min walk on the instrumented walkway in the RE followed each virtual environment condition to washout possible residual effects of walking in the virtual environment. Older adults walked 1 min less than younger subjects to reduce the risk of fatigue. Moreover, in our previous study we noticed that most of the adaptations happened in the first 5 min of walking. Subjects could take breaks between sessions as necessary to minimize confounding effects of exhaustion or poor effort on gait. A virtual reality headset (HTC VIVE) was worn during all conditions in the virtual environment. A Unity3D program was created to display a virtual walkway within the VR headset, with the virtual walkway calibrated to align with the physical walkway in the RE. The 3D virtual scene was set outside with trees and grass along the pathway. Two stationary cameras were used to track the position and orientation of the VR headset in space. The VR headset cables were connected to the laptop containing the virtual scene and the laptop was placed in a backpack worn by a researcher as the subject walked on the walkway to prevent tangling of the wires. Subjects had a first-person view of themselves during walking trials in the VE, as their bodies were not rendered in the VE. To account for the possibility of learning effects, the order in which subjects experienced AP and ML visual perturbations was randomized, with some subjects experiencing AP followed by ML perturbations and vice versa. Randomization was not performed for the RE and VE conditions in accordance to the protocol followed in Martelli et al.^18^. We wanted the participants to get used to the unperturbed VE before starting with the AP and ML conditions in order to ensure that gait modifications were mostly due to the visual perturbations rather than the VE itself. Although subjects were informed that they might encounter AP or ML visual perturbations, they were not aware of the magnitude or direction of the perturbations. Perturbations were presented to subjects by superimposing oscillations of the visual field on normal visual flow; oscillations consisted of a pseudo-random sum of sines of four different frequencies using the equation:

$$ {\text{D(t)}} = {\text{A}}[{\sin}(0.{16} \cdot {2}\uppi {\text{t}}) + 0.{\text{8sin}}(0.{21} \cdot {2}\uppi {\text{t}}) + {1}.{\text{4sin}}(0.{24} \cdot {2}\uppi {\text{t}}) + 0.{\text{5sin}}(0.{49} \cdot {2}\uppi {\text{t}})] $$

where D(t) represents the superimposed displacement of objects in meters that subjects perceive during visual perturbations in the VE, A represents a scaling factor of 0.5, and t represents time in seconds^18^.

PKMAS gait recording and analysis software was used to measure spatiotemporal gait parameters at 120 Hz from the instrumented walkway. Specifically, stride length (SL), stride width (SW), stride time (ST), and stride velocity (SV) were calculated for both the left and right feet. Here, SL is defined as the distance between successive heel strikes of the same foot in the direction of gait progression, SW is defined as the perpendicular distance between a line connecting two ipsilateral heel strikes and the contralateral heel strike, stride time is calculated as the time between consecutive heel strikes of the same foot, and stride velocity is stride length/stride time. These spatio-temporal parameters were selected as they were analyzed in many similar studies^22,24,29,30^ and showed to be the most meaningful ones that we were able to extract with PKMAS software.

The VR system is only able to track a limited area, which restricted the length a subject could walk continuously in the hallway. The VR working space was only slightly longer than the walkway (with a total length of 6.6 m, including both sensorized and non-sensorized areas). In order to ensure that the steps used in the analysis were collected during steady state walking, for each lap the first and last three steps were excluded from the analysis. Furthermore, (1) the first and last steps were outside the sensorized area of the walkway and were not recorded; and (t) the second and penultimate steps were inside the sensorized area of the walkway but were excluded from the analysis. Accordingly, gait recordings started at the end of the third step (i.e., the first analyzed stride was based on the fourth and fifth steps). Moreover, data processing excluded data points if subjects stepped off the walkway or if footprints were unrecognizable by the software or manually. Fifteen random strides for each minute of walking taken in the middle of the walkway were analyzed in each walking condition. This was the maximum number of strides that would allow for each subject in each condition and minute of walking to have the same number of strides to be included in gait analysis.

Mean and standard deviation values were calculated for each gait parameter (SL, ST, SW, SV), where variability in each parameter is represented by the coefficient of variation ([standard deviation/mean] ⋅ 100) expressed as a percentage of the mean. Thus, SLV is stride length variability, SWV is stride width variability, STV is stride time variability, and SVV is stride velocity variability. Increased gait variability has been associated with an increased risk of future falls, even when the systems that contribute to gait variability only show subtle decline^31^. Thus, gait variability as well as other spatiotemporal gait parameters assessed in the VE may be particularly important for early identification of gait abnormalities in the elderly and for therapy^24^.

Data was analyzed using mixed design 3-way repeated-measures ANOVAs (rmANOVAs) to assess main and simple interaction effects of group (two levels: elderly and young), condition (four levels: RE, VE, AP, and ML) and time (two levels: minutes 1and 5) on gait variables. For analysis, group was the only between-subject variable. If significant, main and interaction effects were followed with Tukey’s Honest Significant tests. The Lilliefors, and Mauchly’s tests were performed to check the normality and sphericity assumptions of data. The Huynh–Feldt correction was applied if data violated the sphericity condition. Statistical significance was set at *p* < 0.05.

## Results

**Figure 1:**
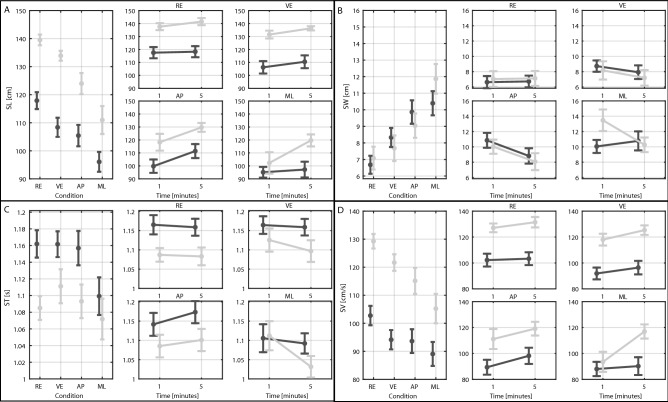
Results: Mean gait parameters. (**A**) Stride Length (SL). (**B**) Stride Width (SW). (**C**) Stride Time (ST). (**D**) Stride Velocity (SV). On the left graphs each point represents the mean value for the young (light grey) and older (dark grey) participants among the first and fifth minutes of walking in the real environment (RE), virtual environment (VE), Medio-Lateral perturbations (ML) and Antero-Posterior perturbations (AP). The right smaller graphs represent the effect of time for each group and condition. Error bars refer to standard errors.

**Figure 2:**
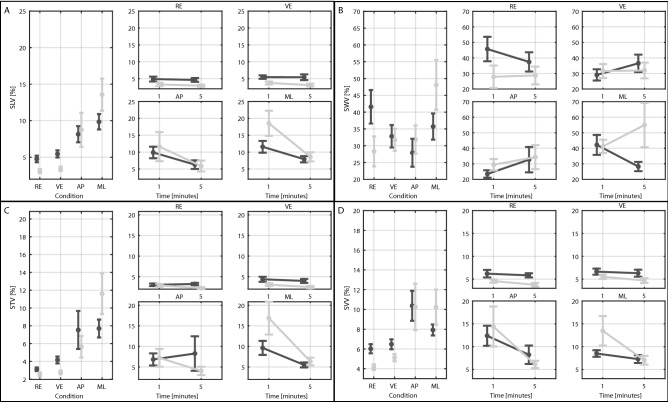
Results: Gait variability parameters. (**A**) Stride Length Variability (SLV). (**B**) Stride Width Variability (SWV). (**C**) Stride Time Variability (STV). (**D**) Stride Velocity Variability (SVV). On the left graphs each point represents the mean value for the young (light grey) and older (dark grey) participants among the first and fifth minutes of walking in the real environment (RE), virtual environment (VE), Medio-Lateral perturbations (ML) and Antero-Posterior perturbations (AP). The right smaller graphs represent the effect of time for each group and condition. Error bars refer to standard errors.

Figures 1 and 2 show spatiotemporal gait parameters (SL, SW, ST, SV) and their variabilities (SLV, SWV, STV, SVV) for each group (E and Y), condition (RE, VE, AP, and ML) and minute of walking (1, and 5). Tables 2 and 3 report the results of the statistical analysis.

**Table 2:** Results of the mixed design 3-way repeated-measures ANOVAs.

3-way rmANOVA	DF	Spatiotemporal gait outcome variables *p* values (F-values)							
		SL	SLV	SW	SWV	ST	STV	SV	SVV
Group	1	**0.011** **(7.79)**	0.284 (1.21)	0.889 (0.02)	0.907 (0.02)	0.315 (1.06)	0.095 (3.04)	**0.018** **(6.49)**	0.253 (1.38)
Condition	3	** < 0.001** **(33.81)**	** < 0.001** **(14.09)**	** < 0.001** **(36.34)**	**0.034** **(3.19)**	**0.029** **(3.84)**	** < 0.001** **(19.45)**	** < 0.001** **(17.09)**	**0.002** **(6.49)**
Time	2	** < 0.001** **(32.52)**	**0.001** **(13.06)**	**0.002** **(12.12)**	0.900 (0.02)	0.159 (2.13)	** < 0.001** **(15.79)**	** < 0.001** **(20.19)**	**0.001** **(13.31)**
Group × Condition	3	0.139 (2.06)	0.068 (2.77)	0.130 (2.15)	**0.026** **(3.53)**	0.258 (1.40)	0.127 (2.31)	**0.035** **(3.15)**	0.063 (2.81)
Group × Time	2	0.145 (2.28)	0.125 (2.54)	0.124 (2.55)	0.094 (3.11)	0.165 (2.06)	0.118 (2.65)	0.088 (3.18)	**0.048** **(4.36)**
Condition × Time	6	0.075 (2.68)	**0.016** **(4.76)**	**0.037** **(3.19)**	0.438 (0.88)	**0.043** **(3.62)**	**0.002** **(8.24)**	0.093 (2.59)	0.089 (2.58)

Factors: Group (two levels: elderly (E) and young (Y)), Condition (four levels: Real Environment (RE), Virtual Environment (VE), Medio-lateral Perturbations (ML) and Antero-posterior Perturbations (AP)) and Time (two levels: minutes 1and 5). Spatiotemporal Gait Outcome Variables: Stride Length (SL), Stride Length Variability (SLV), Stride Width (SW), Stride Width Variability (SWV), Stride Time (ST), Stride Time Variability (STV), Stride Velocity (SV) and Stride Velocity Variability (SVV). The Huynh–Feldt correction was applied if data violated the sphericity condition. Statistically significant *p* values (*p* < 0.05) are bolded. F-values are reported in parenthesis. DF: Degrees of Freedom.

**Table 3:** Results of the Tukey’s honest significant tests.

Tukey’s Honest significant tests		Spatiotemporal gait outcome variables *p* values							
		SL	SLV	SW	SWV	ST	STV	SV	SVV
Post type	VE-RE	**0.002**	0.974	**0.005**	0.959	0.965	0.561	**0.013**	0.961
	ML-VE	** < 0.001**	** < 0.001**	** < 0.001**	0.158	0.143	** < 0.001**	**0.002**	**0.007**
	AP-VE	0.079	0.121	**0.031**	0.549	0.439	0.060	0.684	0.073
	AP-ML	** < 0.001**	0.103	** < 0.001**	**0.014**	0.266	**0.010**	**0.010**	0.996
Group × Type	Y: VE-RE	–	–	–	0.941	–	–	**0.038**	–
	Y: ML-VE	–	–	–	**0.049**	–	–	**0.002**	–
	Y: AP-VE	–	–	–	1.000	–	–	0.130	–
	Y: AP-ML	–	–	–	**0.032**	–	–	0.059	–
	E: VE-RE	–	–	–	0.567	–	–	0.270	–
	E: ML-VE	–	–	–	0.994	–	–	0.375	–
	E: AP-VE	–	–	–	0.233	–	–	0.889	–
	E: AP-ML	–	–	–	0.322	–	–	0.131	–
Condition × Time	RE: 1 versus 5	–	0.952	0.880	–	0.103	0.069	–	–
	VE: 1 versus 5	–	0.446	**0.009**	–	0.149	0.425	–	–
	AP: 1 versus 5	–	0.130	** < 0.001**	–	0.064	0.112	–	–
	ML: 1 versus 5	–	** < 0.001**	0.099	–	0.094	**0.001**	–	–
Group × Time	Y: 1 versus 5	–	–	–	–	–	–	–	** < 0.001**
	E: 1 versus 5	–	–	–	–	–	–	–	0.282

Factors: Group (two levels: elderly (E) and young (Y)), Condition (four levels: Real Environment (RE), Virtual Environment (VE), Medio-lateral Perturbations (ML) and Antero-posterior Perturbations (AP)) and Time (two levels: minutes 1and 5). Spatiotemporal Gait Outcome Variables: Stride Length (SL), Stride Length Variability (SLV), Stride Width (SW), Stride Width Variability (SWV), Stride Time (ST), Stride Time Variability (STV), Stride Velocity (SV) and Stride Velocity Variability (SVV). Statistically significant *p* values (*p* < 0.05) are bolded.

SL and SV showed a significant main effect of group. Older adults walked with a shorter SL (E: 107.6 ± 21.8 cm; Y: 127.1 ± 19.6 cm, *p* = 0.011, Fig. 1A) and slower SV (E: 95.3 ± 23.2 cm; Y: 117.8 ± 21.3 cm, *p* = 0.018, Fig. 1A) than young adults.

All outcomes showed significant main effects of condition (*p* < 0.034). SWV and SV showed a significant group × condition interaction term (*p* < 0.035). While walking in the VE, participants took significantly shorter (VE: 117.9 ± 21.7 cm; RE: 126.0 ± 19.1 cm, *p* = 0.002, Fig. 1A) and wider (VE: 8.1 ± 3.7 cm; RE: 6.8 ± 3.4 cm, *p* = 0.005, Fig. 1B) strides than in the RE. While walking in the VE, only young adults took slower strides than in the RE (VE: 121.6 ± 14.5 cm/s; RE: 129.2 ± 12.7 cm/s, *p* = 0.038, Fig. 1D). During AP perturbations, participants took wider (AP: 9.5 ± 3.9 cm; VE: 8.1 ± 3.7 cm, *p* = 0.031, Fig. 1B) strides than unperturbed walking (i.e., VE).

During ML perturbations, participants took significantly shorter (ML: 102.4 ± 23.0 cm; VE: 117.9 ± 21.7 cm, *p* < 0.001, Fig. 1A), wider (ML: 11.0 ± 4.2 cm; VE: 8.1 ± 3.7 cm, *p* < 0.001, Fig. 1B), and slower strides (ML: 96.0 ± 25.9 cm/s; VE: 104.5 ± 23.4 cm/s, *p* < 0.001, Fig. 1B) with higher SLV (ML: 11.4 ± 8.5%; VE: 4.7 ± 2.8%, *p* < 0.001, Fig. 2A), STV (ML: 9.4 ± 8.6%; VE: 3.6 ± 2.1%, *p* < 0.001, Fig. 2C) and SVV (ML: 8.9 ± 6.2%; VE: 6.0 ± 2.8%, *p* = 0.007, Fig. 2D) than unperturbed walking (i.e., VE).

ML perturbations caused greater reductions in SL (*p* < 0.001, Fig. 1A) and SV (*p* = 0.010, Fig. 1D) and increments in SW (*p* < 0.001, Fig. 2B), SWV (*p* = 0.014, Fig. 2B), and STV (*p* = 0.010, Fig. 2C) than AP perturbations.

Surprisingly, SWV only increased significantly in the younger participants during ML perturbations with respect to the VE (*p* = 0.049) and AP (*p* = 0.032) conditions. Despite this, no differences in SWV were found between younger and elders in any walking conditions (*p* > 0.195). SV only significantly decreased in younger participants from VE to ML (*p* = 0.002). As a result, significant differences in stride velocity were found in the RE and VE conditions (*p* < 0.007).

Except for ST and SWV, all outcomes exhibited significant main effects of time (*p* < 0.002). Only SVV showed a significant group × time interaction (*p* = 0.048). SW, ST, SLV, and STV showed a significant condition × time (*p* < 0.043). Over time, participants took longer (SL—Minute 1: 112.0 ± 23.5 cm; Minute 5: 118.8 ± 22.0 cm, *p* < 0.001, Fig. 1A), and faster (SV—Minute 1: 100.8 ± 24.8 cm/s; Minute 5: 107.8 ± 24.8 cm/s, *p* < 0.001, Fig. 1D) strides with lower SVV (Minute 1: 8.7 ± 7.7%; Minute 5: 6.2 ± 4.0%, *p* = 0.001, Fig. 2D). Further analysis revealed that: (1) both groups reduced SLV (Minute 1: 14.4 ± 10.3%; Minute 5: 8.2 ± 4.0%, *p* < 0.001, Fig. 2A) and STV (Minute 1: 12.6 ± 10.7%; Minute 5: 5.9 ± 2.7%, *p* = 0.001, Fig. 2A) over time only during the ML perturbations; and (2) narrower SW over time only during VE (Minute 1: 8.5 ± 3.6 cm; Minute 5: 7.7 ± 3.8 cm, *p* = 0.009, Fig. 1B) and AP (SW—Minute 1: 10.5 ± 3.7 cm; Minute 5: 8.5 ± 3.9 cm, *p* = 0.001, Fig. 1B) conditions.

## Discussion

In this study, we sought to utilize a virtual reality headset to analyze age-related differences in gait patterns during overground walking when exposed to the virtual environment and to visual perturbations, and to understand to what extent older adults are able to modify their gait over time in response to visual perturbations. To the best of our knowledge, this is the first study examining these age-related differences utilizing continuous oscillations of the visual field during overground walking with a virtual reality headset.

As expected, we found that the VE was associated with decreased stride length and stride velocity and increased stride width in both older and younger adults when compared to the RE^18,20,23^. The introduction of visual perturbations posed a gait challenge in both groups. Specifically, AP perturbations were associated with increased SW and ML perturbations were also associated with decreased SL and SV. Exposure to visual perturbations increased variability in SL, ST, and SV with ML perturbations^18,24–26^. Moreover, ML perturbations caused further reductions in SL and SV and greater increases in SW, SWV, and STV than AP perturbations. We expected this anisotropic response and it is in line with previous studies^10,18,22^. It is inherently more challenging to maintain walking balance in the ML direction than it is in the AP direction^32^. Walking requires step-by-step, integrative control as it is passively unstable in the ML direction, whereas balance is passively stabilized with a series of controlled falls in the AP direction^10^. Moreover, the AP disturbances visually superimposed a velocity to the forward walking velocity whereas ML perturbations superimposed a sideway velocity in a direction orthogonal to walking. Thus, AP perturbation could be less perceivable than ML perturbation^22^.

Our hypothesis that the spatiotemporal gait characteristics of older adults would be more affected by the VE and visual perturbations than younger adults was not supported by the results. Despite the shorter stride lengths and slower stride speeds than their younger counterparts in all conditions^25^, the older adults seemed less affected by visual perturbations, especially in the ML direction than the young participants. While older adults maintained a slow walking velocity for all conditions, young participants significantly slowed down during the visual perturbations. Young adults also showed a larger increase in SWV with ML perturbations than old adults, in contrast to previous findings^24,25^.

These results are intriguing since it is well known that older adults are more reliant on visual feedback for balance regulation and locomotion^14,15^ and could indicate that this effect may be context-dependent^11^. The study by Francis et al.^24^ was done on a treadmill with a fixed walking speed such that participants were not able to fully modulate their gait speed. Additionally, while we found significant differences in walking speed and stride length between young and older subjects, with younger adults walking with longer strides in all conditions, Francis et al. did not find a significant difference in normalized walking speed between age groups. Thus, this discordance of results may be related to younger adults’ faster walking speed in this study making them more susceptible to perturbations. Indeed, Stokes et al. hypothesized that walking slower would allow more time to make kinematic adjustments to preserve balance when exposed to visual perturbations, thus leading to smaller increases in kinematic variability^30^. In other words, walking at slower speeds could benefit anticipatory strategies, such as increased step width, with a decreased need for reactive adjustments in steps^30^. Indeed, they found that increased walking speed was associated with increased step width variability when subjects were subjected to ML visual oscillations of the visual field^30^. This is in accordance with our results showing that younger adults, who walked faster than older adults, showed greater increases in stride width variability in the ML condition. In light of our findings, it’s possible that older adults may walk slower as a general anticipatory strategy to maintain balance while younger adults, more confident in their faster and stronger reactive responses^33,34^, preferred to walk faster, even if this resulted in an increase in SWV and a general need for greater reactive adjustments to maintain balance.

Another reason to explain our results could be found in the amplitude of perturbation and the type of VR system used in this study. While previous studies have found that older participants are usually more affected by visual perturbations, these differences were not seen for higher amplitudes of visual oscillations. Indeed, Qiao et al.^25^ showed similar step width and length variabilities between young and older subjects walking at the largest amplitude perturbation of 50 cm, the same amplitude that was used in this study. Moreover, to the best of our knowledge, this is the first study that analyzed the age-related effects of continuous visual oscillations using a head mounted display that allows for a fully immersive experience with a first-person view and no rendering of body parts. All previous experiments used screens or dome^14,16,24–26^ thus allowing users to see both the moving VR screen and their body while standing or walking on a treadmill that was not moving. Since older adults rely more on visual feedback., this may create a greater visual conflict with respect to our condition in which all the visual field seen by the user is moving in the same way. In addition, older adults have lower perceptions of moving visual stimuli^11^, which could have reduced the effect of optic flow cues in a fully-immersive VR headset. Indeed, it has been shown that older adults are not able to adjust their locomotor patterns in response to a change in the direction of optic flow and still maintain straight walking trajectories in a VR headset^11^. Given the higher average age of our sample, the level of visual perception of our participants could have been even lower with respect to younger elders that participated in previous studies^14,16,25–27^.

Our second hypothesis was also not confirmed by the results. Older and younger adults were able to adapt similarly to the VE and visual perturbations. Both groups showed a reduction of stride length and stride time variability and a progressive shift of average stride length, stride width and stride velocity toward values seen in normal unperturbed walking^18^. The only exception was for stride velocity variability such that only young participants reduced theirs over time. These findings are in partial contradiction with prior studies suggesting that older adults generally adapt less quickly and/or completely to changing or new visual information^11,13,15–17^. However, even though some of these studies showed lower rate of sensory reweighting over time, they also showed that young, healthy, and fall-prone older adults are able to decrease gain over multiple repetitions of the same trial in a similar way^16^. This suggests a capability of adaptation to the visual stimulus movement, which is independent of age or fall-prone status and could be beneficial to implement preventive and rehabilitation programs targeting postural control in older adults^35^. Even with a few minutes of full immersion in VE and exposure to sensory conflicts in the form of pseudorandom oscillations of the visual field, it seems possible for the central nervous system to recalibrate and adapt to the changes and improve walking balance in older adults. VE training under conditions of sensory conflicts could be considered as a potential strategy to reduce the risk of falling^23,35^. An over-ground gait training paradigm with continuous visual perturbations could be used to train subjects to refine the relative weighting of different sensory feedbacks, through the exposure to visual perturbation in a more ecological condition that is not affected by the limitations of treadmill walking. While mechanical perturbations could generate similar^22^ or greater^36^ disruptive changes in gait and balance, visual perturbations allow to still affect balance without physically applying forces to the body that may increase the risk of injury, especially for more frail elders. Moreover, visual perturbations delivered by commercially available VR headsets, like the one used in this study, could have multiple benefits. Not only are they more economical from a cost perspective but they are a relatively simple set up that can be used at home thus promoting remote monitoring of balance that can alleviate the burden of traveling to the clinic. Future studies should focus on whether such adaptations in the virtual environment can be generalized to the real environment^37^ and whether a training program of longer duration could generate long-term effects.

There are several important limitations to this study. The walkway and the working space of the VR headset were small, which necessitated that subjects make multiple turns within each condition, rather than having continual step progress. Moreover, it limited the amount of space for the acceleration and deceleration phases. Despite this, we took the precaution to analyze data starting from the end of the third step. Considering that the average stride length among all subjects and conditions was 1.17 m, an average of 1.76 m of gait initiation and termination were excluded from the analysis at the beginning and end of each lap. As suggested by the guidelines for clinical applications of spatio-temporal gait analysis in older adults from the GAITRite Network group^38^, it is good practice to start and end the recordings at 2 m or 2 full strides (i.e., 4 steps) from the beginning and ending of the walking passes. However, even if we used 3 steps for acceleration/deceleration phases that were in average shorter than 2 m, we believe this was not a major methodological limitation of this study. Indeed, more recent guidelines for the assessment of gait in older adults from the Biomathics and Canadian Gait Consortiums Initiative reported that a steady-state gait can be achieved by instructing participants to start walking 1 m prior to the data recording zone and stopping at least 1 m beyond it^39^. Moreover, other studies have suggested that the transition of the body to steady gait pattern occurs rapidly over a period that can range from one^40^ to three steps^41^ and that measures of gait variability measured on a shorter walkway with shorter acceleration/deceleration phases (i.e., 4-m walkway plus 1-m for acceleration and deceleration phases) showed: (1) good test–retest reliability and concurrent validity, as indicated by associations with functional status and poorer health^42^; (2) associations with history of falls in the past year^43^; and (3) predictive ability for future mobility impairments and disability^44^.

Simple spatio-temporal metrics of gait derived solely from kinematic measures were used to analyze data. These metrics may not fully capture the complexities of dynamic balance control. Future experiments could use (1) larger working areas thanks to the wider tracking field of new versions of the device (10 m × 10 m) and (2) data recorded by motion trackers affixed on the user to record continuous position, velocity and accelerations of different body segments, thus allowing to extrapolate more complex measures of gait stability that could better reflect the subjects ability to control balance. Additionally, a longer time period of walking in each condition would allow for better understanding of the completeness of gait adjustments by elderly individuals when exposed to perturbations. As the subjects in this experiment were generally healthy elderly adults without diagnosed gait or balance problems, this study cannot be generalized to the overall population of elderly adults. Future studies should focus on fall-prone older adults with diagnosed balance or vertigo problems. Findings from this study help to further characterize gait in the elderly under normal and perturbed walking conditions and suggest an ability to adapt to virtual challenges.

## Data Availability

The datasets analyzed during the current study are available from the corresponding author on request.
